# Advancing AAV vector manufacturing: challenges, innovations, and future directions for gene therapy

**DOI:** 10.3389/fmmed.2025.1709095

**Published:** 2025-12-03

**Authors:** N. Charan S. S. Kowshik, Pushpendra Singh

**Affiliations:** 1 Ocugen India, Hyderabad, Telangana, India; 2 Ocugen, Malvern, PA, United States

**Keywords:** aav, viral vectors, gene therapy, manufacturing, process characterization, process validation

## Abstract

Adeno-Associated Virus (AAV) vectors are at the forefront of gene therapy, offering transformative therapeutic potential for many genetic disorders. However, the translation of this promise into accessible treatments is constrained by manufacturing challenges, including process variability, low yields, and scalability challenges. This review provides a comprehensive framework for establishing robust AAV-based gene therapy manufacturing processes by evaluating industry challenges and recent technological innovations. We studied the end-to-end AAV-based gene therapy manufacturing process, from upstream unit operations such as cell culture and transfection to downstream purification and fill-finish operations. Key upstream innovations highlighted include high-density perfusion cultures, advanced single- and dual-plasmid systems, and next-generation transfection reagents that collectively enhance the overall process quality and viral vector productivity. In the realm of downstream processing, recent advancements in serotype-agnostic affinity chromatography and ion-exchange chromatographic purifications have enhanced the critical separation of full capsids from empty capsids. The implementation of a quality-by-design framework is the heart of the AAV-based gene therapy manufacturing process. We emphasize the necessity of a rigorous process characterization, utilizing validated scale-down models and design of experiments, as a prerequisite for establishing a robust control strategy with defined proven and normal operating ranges. This data-driven approach not only mitigates process inconsistency, but it also serves as the foundation for an effective process validation and regulatory compliance. Looking ahead, the integration of artificial intelligence and continuous manufacturing methodologies will be pivotal in expediting the development of safer, more efficacious, and personalized AAV-based gene therapies.

## 1 Introduction

### 1.1 Background

More than 10,000 rare disorders were identified and described worldwide. Among them, 80% are genetic in origin. Nearly 10% of the population is affected by 8,000 rare genetic disorders, making it a public health concern (Byrne et al., 2025). Even after exponential progress and efforts made in the field of gene therapy, only 5% of these patients have treatment options for their medical condition (Drag et al., 2023; Byrne et al., 2025). Among the available gene delivery methods, AAV-mediated gene delivery is superior as it offers targeted gene delivery with broad tissue tropism, favorable safety profile and durable transgene expression (Li and Samulski, 2020; Meier et al., 2020; Miller et al., 2020; Mendell et al., 2021; Drag et al., 2023; Byrne et al., 2025). The use of recombinant AAVs in gene therapies has gained significant attention owing to their favorable safety and efficacy profile, highlighting the importance of strategic vector design, dose optimization, and long-term safety monitoring (Angela et al., 2023; Wang et al., 2024). Some serious adverse events associated with AAV-based gene therapies have been reported at very high doses (Angela et al., 2023; Duan, 2023); However, a direct causality is difficult to establish, as no antibodies against the AAV capsid or transgene were detected, and the events were attributed to a non-specific innate immune response. Overall, AAV-based gene therapies remain considered safe due to their non-integrating nature and low immunogenicity (Wang et al., 2024). Different AAV-based gene therapies have demonstrated their therapeutic potential as an ideal carrier in transporting therapeutic genes into specific cells and leading the way for innovative treatments for many genetic disorders such as spinal muscular atrophy and Leber congenital amaurosis (Russell et al., 2017; Ozelo et al., 2022; Strauss et al., 2022; Simons et al., 2023; Pipe et al., 2023; Adam et al., 2024; Wang et al., 2024; Mendell et al., 2025). This surge in approval, growing demand, better safety and efficacy profile of AAV-based gene therapies can be harnessed in personalized medicine to create tailor-made treatments for patients with rare, ultra-rare conditions (Kiran et al., 2025). The list of approved AAV-based gene therapies till date is provided in Table 1 (U.S. Food and Drug Administration, 2025).

**TABLE 1:** List of approved AAV-based gene therapies.

Products	Luxturna	Zolgensma	Bequez	Kebilidi	Elevidys	Hemgenix	Roctavian
AAV serotype	AAV2	AAV9	AAVRh74var	AAV2	AAVrh74	AAV5	AAV5
Sponsor	Spark therapeutics	Novartis gene therapies	Pfizer	PTC therapeutics	Sarapeta therapeutics	CSL Behring LLC	BioMarin Pharmaceuticals
Generic name	Voretigene neparvovec	Onasemnogene abeparvovec	Fidanacogene elaparvovec	Eladocagene exuparvovec	Delandistrogene moxeparvovec	Etranacogene dezaparvovec	Valoctocogene roxaparvovec
Gene Delivered	RPE65 gene	SMN1 gene	F9 gene	Dopa Decarboxylase gene	Dystrophin gene	F9 gene	F8 gene
Administration route	Subretinal injection	Intravenous infusion	Intravenous infusion	Intracerebral injection	Intravenous infusion	Intravenous infusion	Intravenous infusion
Indication	RPE65-mediated inherited retinal dystrophy	Spinal muscular atrophy type 1	Severe hemophilia B	Aromatic L-amino acid decarboxylase deficiency	Duchenne muscular dystrophy	Hemophilia B	Severe Hemophilia A
US FDA approval year	2017	2019	2024	2024	2023	2022	2023

**FIGURE 1:**
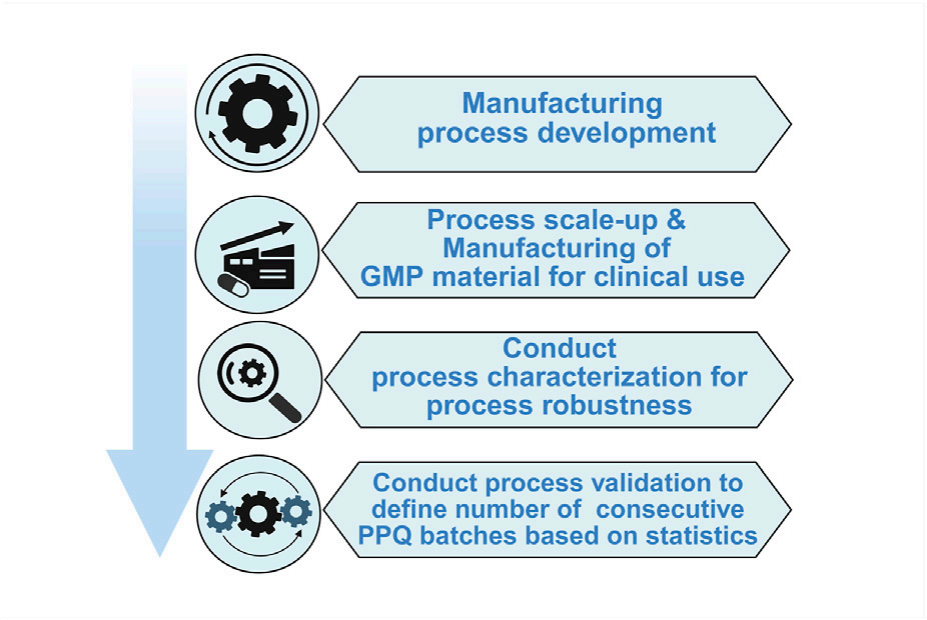
General workflow of AAV vector manufacturing shows sequential unit operations across the production continuum. This schematic outlines the major stages of AAV production, color-coded by function, upstream unit operations (red) such as cell culture & plasmid transfection; downstream unit operations (green) including lysis, chromatography, and sterile filtration; and fill–finish operations (blue) representing formulation, sterile filtration, and final container filling and storage. The figure emphasizes the connectivity between process segments and their collective impact on final product quality.

The cost of development of AAV-based gene therapies is quite high, and it would be improbable to manufacture therapy for each genetic disease if we follow the traditional manufacturing route from development to commercialization as described in Figure 1.

The complexity and novelty of AAV-based gene therapies present unique challenges in developing and establishing robust manufacturing processes and meeting regulatory expectations. Accelerated development is impeded by the need for extensive process optimization (developmental batches, scale up for good laboratory/good manufacturing practice campaigns and process characterization), analytical testing and product stability data to address unmet medical needs. The current review provides a framework to establish well optimized and validated manufacturing processes for AAV-based gene therapies, while addressing industry-wide challenges and recent advancements.

### 1.2 Manufacturing process description

**FIGURE 2:**
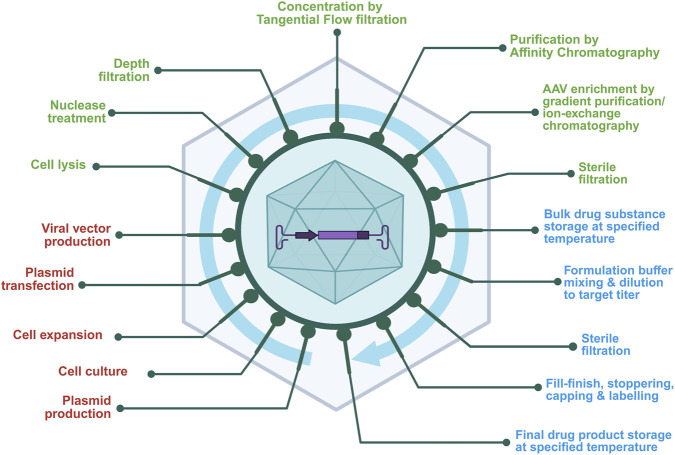
General workflow of AAV vector manufacturing shows sequential unit operations across the production continuum. This schematic outlines the major stages of AAV production, color-coded by function, upstream unit operations (red) such as cell culture and plasmid transfection; downstream unit operations (green) including lysis, chromatography, and sterile filtration; and fill–finish operations (blue) representing formulation, sterile filtration, and final container filling and storage. The figure emphasizes the connectivity between process segments and their collective impact on final product quality.

The AAV-based gene therapy manufacturing involves multi-step processes (Figure 2) designed to ensure efficient production and high-quality viral vectors for gene therapy. A strategic approach is essential to meet clinical and regulatory requirements, combining traditional approaches with novel technologies to ensure product quality, consistency, and patient safety.

As described in Figure 2, the manufacturing process begins with large-scale plasmid production and purification. Traditionally three plasmids are prepared for transfection.

- A cis acting plasmid carrying therapeutic gene.
- A trans-acting plasmid with genes for AAV replication and capsid formation.
- Another trans-acting plasmid enabling AAV replication in the host cells.

These plasmids are transfected into AAV-producing cells (e.g., human embryonic kidney HEK293 cells), from master/working cell banks are thawed, cultured and expanded and then inoculated into the bioreactor for large-scale production. The plasmid transfection is conducted in the bioreactor, and after 2–3 days, the cells are harvested for the downstream purification.

In downstream purification, the cells are lysed to release AAV vectors into the culture medium and then nuclease treatment is performed to degrade the host cell DNA. Then the purification process comprising ultracentrifugation, chromatography, and ultrafiltration (UF) and diafiltration (DF) are performed to remove cellular debris and impurities such as plasmid DNA, host cell proteins and host cell DNA, and empty capsids. The in-process testing implemented ensures quality and regulatory compliance by assessing safety, identity, strength, purity and quality (SISPQ) as per regulatory expectations for the viral vectors. The tests include detection of viral contamination, residual DNA/proteins, and vector genome determination. The final steps include formulation, fill-finish and storage and shipment of the drug product (Srivastava et al., 2021; Lee and Chang, 2024).

### 1.3 Challenges and recent advancements in AAV-based gene therapy manufacturing processes

Given the complexity of AAV-based gene therapy manufacturing, a risk-based, science-driven approach is needed to meet safety and efficacy standards, clinical and market demands, and cost of goods. Key challenges include ensuring viral vector stability, preventing vector degradation during manufacturing, handling, and storage, and maintain long-term stability and efficacy. To address patient needs, evolving market and regulatory expectations, a combination of traditional approaches and innovative technologies are needed to develop scalable and robust manufacturing processes for gene therapy products (Srivastava et al., 2021).

#### 1.3.1 Upstream processing steps

##### 1.3.1.1 Cell culture

Cell culture is the early step in viral vector manufacturing where cells are cultured to multiply in a large bioreactor to attain a required cell density in specific growth conditions (Figure 2). There are different options available for performing cell culture. Each method offers distinct advantages and limitations of their own. Hence, the choice of the technique depends on the cell type, research goals, and scalability requirements (Srivastava et al., 2021).

In adherent cell culture, cells grow attached to a solid surface, such as a flask or plate whereas in suspension culture, cells grow freely within the culture medium without requiring surface attachment (Srivastava et al., 2021; Cytiva Lifesciences, 2025b).

The primary challenge lies in deciding on the most suitable technique in terms of scalability and cost of production. The adherent culture requires more operator intervention and leads to higher risk of contamination. The expansion of seed trains is more labor-intensive, and space demanding and requires flatware or specialized bioreactors (e.g., hyper stack cell culture vessels) increasing equipment and resource needs (Srivastava et al., 2021; Cytiva Lifesciences, 2025b).

Suspension cultures, in contrast, are generally easier to scale and operate. They eliminate the need for dissociating cells from the surfaces during passage, scale up, counting thus simplifying the workflow. These cultures require less hands-on maintenance, less processing time, and less space in incubators and facilitate easier sampling and seeding through pumps or syringes (Srivastava et al., 2021; Cytiva Lifesciences, 2025b). Nonetheless, media exchanges are more complex in the suspension culture, due to free floating nature of the cells, media exchanges are difficult, and perfusion processes require additional equipment.

The manufacturing space has been evolving in terms of continuous research with day-to-day improvements which can overcome the manufacturing hurdles. Some of the notable advancements made in cell culture were reported as follows:

For adherent systems, the use of single-use, scalable fixed-bed perfusion bioreactors (iCELLis by Cytiva) demonstrated superior scalability of the process by providing a controlled environment designed for achieving high cell densities, increased viral yields, and lot-to-lot consistency. The achievement of 8-fold higher productivity by performing post-transfection media exchange and by preventing the recirculation of media improved transfection efficiency (Cytiva Lifesciences, 2024). In case of suspension culture, an N-1 perfusion process was developed for HEK293 suspension cells using Xcellerex APS bioreactor (Cytiva), connected to an Xcellerex XDR-50 bioreactor. Using this setup, a 2000–5000 L AAV vector production bioreactor after a 7-day perfusion, a 35 × 10^6^ cells/mL cell density was achieved with >95% viability (Cytiva Lifesciences, 2025a). Both adherent and suspension cultures offer distinct advantages and should be selected and tailored based on the specific requirements of the manufacturing process.

##### 1.3.1.2 Enhancing genome packaging efficiency: Rep hybrids engineering

Genome packaging efficiency is a critical determinant of AAV vector quality and productivity. It describes how effectively the viral capsids produced during manufacturing are filled with the intended recombinant AAV genome (Tanaka et al., 2020). In simple terms, high packaging efficiency means that most of the viral particles generated actually contain the therapeutic gene of interest, making the final drug product more potent and consistent (Mario et al., 2021; Wang et al., 2024). When this packaging efficiency is low, a large proportion of empty or partially filled capsids were formed which do not contribute to therapeutic efficacy and also complicate downstream purification, reducing the viral genomes per total capsid ratio and may trigger unwanted immune responses in patients. Studies have shown that improving genome packaging efficiency not only increases vector yield but also enhances dose consistency and overall process sustainability (Grieger et al., 2006; Rumachik et al., 2020).

Several factors influence packaging efficiency, including the quality of plasmids, the accuracy of inverted terminal repeat (ITR) sequences, the balance of Rep and Cap protein expression, and the optimization of production conditions such as timing, temperature, and helper function expression (Wright, 2009; Asaad et al., 2023). In most AAV vector production systems, the Rep genes derived from AAV serotype 2 are utilized to package single-stranded recombinant genomes across various serotypes and engineered capsids. However, this approach often leads to suboptimal packaging efficiency, resulting in a higher proportion of empty or partial capsids depending on the serotype. Studies have demonstrated the use of novel hybrid Rep genes, combining serotype-specific Rep sequences with the 3′end of the AAV2 Rep gene, significantly increased the proportion of genome-containing (full) capsids by approximately 2- to 4-fold across all tested non-AAV2 serotypes. This strategy represents a promising advancement for improving AAV full capsid yield (Mario et al., 2021; Wang et al., 2024).

##### 1.3.1.3 Plasmid transfection

Plasmid transfection is a critical step used to introduce the necessary genetic material into the host cells to enable production of the AAV vectors. The variability in transfection efficiency is the key challenge in transfection process. An efficient transfection process is dictated by three key factors: the plasmid system, cell culture condition (cell density and viability), and choice and optimization of transfection reagents. The following key improvements can help researchers to decide on designing a process with enhanced transfection efficiency.

###### 1.3.1.3.1 Tailor-made plasmid design

Traditional triple-plasmid transfection is the fast and efficient way to manufacture AAV. However, triple plasmid often results in inconsistent plasmid uptake by host cells, leading to variation in empty-to-full capsid ratios. To overcome these limitations, advanced single- and dual-plasmid systems containing the required genes are developed to improve transfection efficiency, enhance vector yield and streamline scalability. Below are some notable advancements in plasmid system design.

- Dual-plasmid system: This platform combines transgene, AAV rep/cap, and accessory genes to a single plasmid, with adenoviral helper functions on a second. This configuration significantly increases AAV vector productivity, improves the proportion of full capsids, and maintains high product quality across multiple serotypes and scales, including up to 50-L bioreactors. It outperforms both the traditional triple-plasmid and earlier dual-plasmid systems, offering scalability and flexibility for new capsid variants and genome designs (Moreno Velasquez et al., 2023; van Lieshout et al., 2023).
- AAVone single-plasmid system: The AAVone system consolidates all necessary components (adenoviral helper genes, rep/cap, and transgene) into a single plasmid. This innovation yields a 2 to 4-fold increase in AAV vector production, reduces batch variability, simplifies the process, and lowers DNA impurities, making it especially suitable for GMP-grade vector manufacturing (Yang et al., 2025).

###### 1.3.1.3.2 Key plasmid attributes required for AAV vector production

Plasmids as key starting materials directly influence vector yield, genome integrity, and overall product quality, their design, sequence integrity, and manufacturing under GMP-compliant conditions are critical for ensuring a consistent and safe AAV product suitable for patient safety. The supercoiled nature of plasmids has been considered as a critical quality attribute for transfection resulting in efficient AAV vector production (Tanaka et al., 2020).

- Transfection efficiency and intracellular trafficking: Studies have demonstrated that the plasmids with high supercoiled percentage (SC%) had formed smaller, more compact complexes with transfection reagents, which are taken up by cells more efficiently during endocytosis and this enhanced cellular uptake can lead to a greater number of transfected cells and higher overall protein expression (Cherng et al., 1999; Remaut et al., 2006).
- Product yield and consistency: AAV vector production by transient triple plasmid transfection depends on co-delivery and timely expression of multiple plasmids, any loss of transfection efficiency or heterogeneity between batches (e.g., variable SC%) leads directly to lower or more variable vector titers and impurity profiles, undermining process sustainability. Recent studies have emphasized the importance of plasmid quality and topology as driving factors of rAAV vector titers (Folarin et al., 2019; Pistek et al., 2024).
- Stability and degradation risk: Non-supercoiled plasmid fractions arise from nicking or breakage and are more susceptible to nucleases and shearing during downstream handling and storage, thereby helped in maintaining high SC% reduces the risk of progressive loss of plasmid functionality during manufacturing process (Cai et al., 2010; Hassan et al., 2016).
- Regulatory expectations: Incorporating the plasmid DNA SC% into the bulk release specifications is considered good manufacturing practice, as this attribute directly influences transfection performance and process reproducibility. Setting a defined lower limit commonly above 80% helps maintain consistent AAV vector production outcomes and aligns with regulatory expectations for plasmid quality control (U.S. Food and Drug Administration, 2007).

To ensure robust and compliant AAV vector manufacturing, critical starting materials including plasmids, cell substrates, and other key components must meet stringent quality standards in accordance with regulatory requirements. For optimal bulk plasmid production enriched with a high supercoiled fraction in AAV manufacturing, the process should integrate multiple refinement strategies. Employing recA^−^ and endA^−^ *E. coli* strains minimize plasmid nicking and recombination, thereby enhancing structural integrity (Daegelen et al., 2009; Borja et al., 2012). Cultivation parameters should be carefully optimized by harvesting cells during the logarithmic growth phase, maintaining lower culture temperatures *etc.* Furthermore, precise control over the alkaline lysis and neutralization steps is essential to preserve plasmid integrity, supercoiled DNA suitable for downstream AAV applications (Cronan, 2014).

Downstream purification strategies play a pivotal role in enhancing bulk plasmid production with a high supercoiled DNA fraction for AAV manufacturing. The implementation of anion-exchange chromatography (AEX) or monolith-based purification systems enables effective separation of supercoiled plasmids from open-circular and linear DNA species. Further refinement through dedicated polishing steps—such as optimizing the AEX gradient or incorporating size-exclusion chromatography—can substantially improve the purity and enrichment of the supercoiled fraction, thereby ensuring superior plasmid quality for downstream AAV production processes (Smrekar et al., 2010; Pavlin et al., 2023; Ferreira et al., 2024).

Strategic alternatives such as establishment of stable producer cell lines or adopting baculovirus-mediated AAV expression platforms can substantially reduce the overall plasmid demand, thereby simplifying upstream processing. Additionally, optimizing the ratios of essential plasmids within the transfection mix can further minimize plasmid consumption without compromising vector yield or quality, contributing to a more streamlined and cost-effective production workflow (Wang et al., 2024).

Overall, these approaches combine optimized bacterial growth and purification methods for near-term improvement, while transitioning to stable or baculovirus-based systems offers long-term cost efficiency and scalability. Process optimization represents a low-cost intervention, whereas adding chromatographic polishing steps provides higher purity with moderate added expense. High-quality supercoiled plasmids can also be obtained from reputable CDMOs in large quantities; however, this approach can be costly. Alternatively, plasmid consumption can be reduced by developing stable cell lines that require minimal or no plasmid input, or by adopting plasmid-free baculovirus-based systems for scalable AAV-based gene therapy production.

###### 1.3.1.3.3 Optimized plasmid ratios and plasmid design

The optimization of plasmid ratios using design-of-experiment (DOE) methodology has enabled a data-driven approach to maximize co-transfection efficiency and ensure synchronized expression of vector components, resulting in improved genome packaging and vector titers. In parallel, innovative plasmid designs, including streamlined helper plasmids with reduced non-essential adenoviral elements and minimized backbone size can enhance transfection efficiency. Collectively, these advancements in plasmid ratio optimization and rational plasmid engineering have the ability to transform transient transfection process into a more predictable, scalable, and high-yield platform for AAV vector manufacturing (Park et al., 2024b; van Lieshout et al., 2024).

- DOE optimization: Systematic optimization of the triple-plasmid ratio (pHelper:pRepCap:pAAV-GOI) in HEK293 cells is demonstrated to nearly double genome titers and significantly reduce empty capsid formation for AAV2 and AAV9 serotypes. This approach enhances both the efficiency and quality of AAV vector production (Park S. et al., 2024).
- Improved helper plasmids: Engineered helper plasmids such as OXB-Helper_3 with deletions in adenoviral E4 and E2a genes have led to a >2-fold increase in AAV vector productivity. Their smaller plasmid size facilitates higher yields across multiple serotypes and transfection platforms (van Lieshout et al., 2024).

###### 1.3.1.3.4 High-density and scalable production

Optimizing culture conditions is fundamental in achieving efficient transfection. In traditional batch systems, cell density and viability often decline rapidly after transfection, limiting productivity. In contrast, high-cell-density perfusion cultures maintain cells in an optimal growth phase by continuously supplying fresh nutrients, thereby extending the transfection window and enhancing vector genome replication and packaging efficiency. The controlled environment of perfusion bioreactors enables higher viable cell concentrations and improved plasmid uptake, resulting in superior volumetric productivity. Similarly, advances in suspension culture systems including optimized cell lines, transfection reagents, and feeding strategies have enhanced transfection across large-scale bioreactor volumes. These innovations can collectively improve transfection efficiency, and process scalability, forming the foundation for robust, cost-effective AAV vector manufacturing at clinical and commercial scales (Guan et al., 2022; Deng et al., 2025).

- High-cell-density perfusion culture: Employing high-density perfusion systems and optimizing transfection parameters (plasmid amount, DNA: polyethyleneimine (PEI) ratio, feeding strategies) can achieve unit yields up to 2 × 10^12^ vg/mL, supporting large-scale, cost-effective AAV vector manufacturing for clinical and commercial scales (Deng et al., 2025).
- Suspension culture optimization: Advances in suspension culture using optimized cell lines, transfection reagents, and process parameters have enabled scalable, high-yield AAV vector production suitable for preclinical and research needs (Guan et al., 2022).

###### 1.3.1.3.5 Use of transfection reagents

Among various methods for transfection, calcium phosphate methods are subject to significant batch to batch variability due to reagent purity and pH sensitivity. Liposome transfections are highly efficient and minimally cytotoxic, but reagents are not cost-effective when used for commercial AAV production. PEI is an effective transfection reagent, however it is sensitive to pH and could be toxic to production cells (Srivastava et al., 2021). Once a reagent is selected, transfection conditions need to be optimized for a scalable process for better yield. Transfection reagents with novel chemical modifications have demonstrated enhanced transfection efficiency over PEI under optimized conditions were listed below.

- FectoVIR^®^-AAV (Polyplus-Sartorius) is specifically designed for industrial-scale AAV production in both suspension and adherent HEK293-derived cell systems, reported high rAAV titers and scalability. PEIpro®, particularly its GMP-compliant version, has also been widely adopted for AAV production, with recent advancements focusing on optimizing its performance in HEK-293 suspension cell cultures. As per manufacturer specifications, FectoVIR^®^-AAV can boost AAV vector productivity by 2-fold when compared to PEIpro^®^ (Sartorius), and up to 10-fold compared to other PEI based transfection reagents (Coplan et al., 2024; Polyplus-Sartorius, 2025).
- The TransIT-AAViator Transfection System (Mirus Bio) is a reagent designed to enhance the production of adeno-associated virus (AAV) vectors. It combines a transfection reagent with a separate enhancer (RevIT™ AAV Enhancer), As reported by the manufacturer, this use of this transfection system can achieve higher AAV vector output and improved full/empty capsid ratios, while reducing the amount of plasmid DNA required (Pietersz et al., 2024; Mirus Bio, 2025).
- Hieff Trans™ UltraAAV (Yeasen Bio) - an optimized and modified linear PEI specifically developed for the large-scale production of AAV in suspension systems. The data furnished by manufacturers suggests that using this transfection system reported a substantial increase in virus yield, with approximately a 2-fold increase in virus titer for multiple serotypes, including AAV2, AAV5, AAV8, and AAV9, thereby reducing the amount of transfection reagent by 50% while maintaining a high yield and reducing the production costs (Yeasen Bio, 2025).

Recent innovations by manufacturers in developing high-quality transfection reagents can enhance transfection efficiency by addressing current challenges. This enables researchers to access the available transfection reagents to conduct process development and optimization studies through multiple controls by comparing different transfection reagents across different cell lines. This kind of active research driven environment sets a platform for researchers as well as industry experts to streamline their efforts in the future development of AAV vector manufacturing.

##### 1.3.1.4 Viral infection based AAV vector production

The viral infection-based methods have shown scalability and with high AAV vector yield. However, they suffer from the significant challenges that limit their widespread adoption.

- Baculovirus/insect cells: Genetic instability of the baculovirus and process optimization are required to achieve proper VP1/2/3 ratio (Wang et al., 2024).
- Mammalian stable cell lines expressing Rep/Cap and Ad-AAV hybrid: The development of various HeLa-based producer cell lines present challenges due to process variability and inconsistency. In addition, there is a risk of adenoviral contamination during production (Wang et al., 2024).
- Recombinant Herpes Simplex Virus (rHSV): rHSV-based systems possess the risk of residual HSV contamination, raising safety and regulatory concerns (Wang et al., 2024).
- Tetracycline enabled self-silencing adenovirus (TESSA): While the TESSA system eliminates the need for helper viruses, it offers limited process flexibility and carries the risk of adenoviral contamination (Wang et al., 2024).

The future of AAV vector manufacturing relies on developing producer cell lines that are free from plasmids and do not require transfection or infection. A study reported an enhancement in productivity with synthetic cell lines through data-driven redesign of genetic modules and tuning of viral gene expression for viral vector production platform and its potential for plasmid- and virus-free AAV vector manufacturing (Lu et al., 2024).

#### 1.3.2 Downstream process

##### 1.3.2.1 Cell harvest

The cell harvest process involves collecting and preparing the cells producing viral vectors for downstream processing. Following this, a clarification and filtration step removes cellular debris and larger contaminants to yield a clarified AAV-containing solution. It becomes a challenge manufacturers to handle large cell volumes for cell harvest process, which is even time consuming. Hence, the systems which can combine upstream production with downstream clarification in a single continuous process can be used. The viral harvest unit concept integrates tangential flow filtration systems that allow AAV particles to pass through while retaining cells in the bioreactor. This approach has enabled continuous harvest throughout the production cycle without interrupting cell culture operations (Nguyen et al., 2025).

- Alternating Tangential Flow (ATF) systems with optimized shear rates maintain high cell viabilities while enhancing AAV-specific titers. Research demonstrates that fine-tuning shear rates in ATF systems achieve AAV-specific titers of 7.6 × 10^4^ Vg/cell, representing up to 4-fold improvement compared to non-optimized perfusion cultures. The alternating flow pattern prevents membrane fouling while maintaining continuous harvest capabilities (Mendes et al., 2022b).
- ATF systems enable superior clarification with turbidity reduction below 10 nephelometric turbidity units, though this comes at the cost of lower AAV vector recovery yields compared to TF/DF systems. The sub-micron pore size of ATF membranes provides excellent clarification performance independent of cell concentration at harvest time (Mendes et al., 2022b).

##### 1.3.2.2 Cell lysis

Cell lysis is a critical unit operation in downstream processing that facilitates the release of intracellular viral particles from host cells. The objective of this step is to achieve maximal recovery of intact and infectious AAV capsids while minimizing the co-release of host-derived impurities and avoiding vector degradation. Several lysis methods are employed in AAV manufacturing, including mechanical (freeze–thaw, sonication), chemical (detergent- or surfactant-mediated), and osmotic or high-salt disruption, or combinations thereof. Among these, detergent-assisted, high-salt lysis and in-situ lysis are preferred for commercial applications because they are rapid, scalable, and compatible with downstream clarification and purification processes (Jungbauer and Wheelwright, 2025).

- Detergent-based lysis: This method involves detergents, such as Triton X-100 or biodegradable alternatives, directly added to the cell culture disrupting the cell membranes, thereby releasing the intracellular contents, including the AAV vectors (Jungbauer and Wheelwright, 2025).
- High salt concentration: A high salt concentration in the lysis buffer causes an osmotic shock that can burst the cells and release the AAV vectors. Combining high salt with a salt-tolerant nuclease can increase the yield and infectivity of AAV vectors by preventing aggregation (Jungbauer and Wheelwright, 2025).
- Nucleic acid digestion: As part of the lysis step, an endonuclease (like Benzonase) was often added to digest the host cell DNA and remaining plasmids, which significantly reduced the viscosity of the lysate and facilitates downstream filtration and purification (Jungbauer and Wheelwright, 2025).
- Photochemical lysis: A novel approach where a light-activated photosensitizer was used to target and disrupt the cell membrane. This method has been shown to reduce host-cell impurities compared to standard detergent lysis (Hauge et al., 2025).
- Functional fiber capture: In this technique, cells are captured on functionalized fibers. Lysis is then performed by flushing the fibers with buffer, bursting the cells and releasing the AAV vectors, which are then eluted (Jungbauer and Wheelwright, 2025).

##### 1.3.2.3 Affinity chromatography step

This unit operation in the downstream process offers specific separation based on the interaction between an immobilized ligand and a target molecule where the ligand binds to a protein on the AAV capsid (Danaher Life Sciences, 2025). Affinity chromatography purification helps in concentration of AAV vector particles from other impurities, but this technique does not have the capability to separate the full and empty capsids (Srivastava et al., 2021).

Recent advancements in affinity chromatography for AAV vector purification have focused on improving efficiency, scalability, serotype coverage, and product quality.

- The introduction of serotype-agnostic peptide ligands has enabled broad, high-yield purification across all major AAV serotypes, with milder elution conditions and enhanced resin reusability, addressing limitations of traditional protein-based ligands that require harsh elution and have limited lifespans (Shastry et al., 2024a; Shastry et al., 2024b).
- The POROS CaptureSelect AAVX resin (Thermo Fisher Scientific) has emerged as a versatile platform, efficiently capturing a wide range of divergent AAV serotypes, supporting repeated regeneration without loss of performance, and achieved high recovery rates (65%–80%) at various scales (Florea et al., 2023; Mietzsch et al., 2024).
- Continuous affinity purification using periodic counter-current chromatography has demonstrated stable, high-throughput virus recovery with improved productivity, supporting the shift toward integrated continuous manufacturing (Mendes et al., 2022a).
- Innovations also include the use of affinity-functionalized nanofiber adsorbents, which offered high binding capacity, rapid processing, and reduced pressure drop, potentially streamlining downstream operations (Neto et al., 2023).
- Single-step, semi-automated heparin affinity protocols and dual affinity/ion-exchange workflows further enhanced the purification process, yielding high-purity, infectious AAV vectors suitable for clinical applications (Neto et al., 2023; Lopes et al., 2024).
- Structural studies of affinity ligands, such as AAVX (Thermo Fisher Scientific), have informed capsid engineering strategies to ensure compatibility with purification platforms and maintain broad serotype applicability (Mietzsch et al., 2020; Thakur et al., 2025).

###### 1.3.2.3.1 Purification of full capsids from the empty capsids

Density gradient ultracentrifugation is a conventional technique used to separate and purify AAV vector particles from other cellular components and impurities. It leverages differences in density and sedimentation rates to isolate AAV, including full and empty capsids, and remove contaminants like host cell DNA, RNA, and proteins (Khaparde et al., 2025; Lorek et al., 2025).

Cesium chloride and Iodixanol-based density gradient ultracentrifugation are widely used for the purification of various AAV serotypes. This technique separates target AAV vector particles from contaminants like cellular organelles, soluble proteins, nucleic acids, and non-infectious vectors based on the isopycnic point specific to AAV vectors. The elimination of these contaminants is of paramount importance, as they can induce an immune response and affect the transduction efficiency thereby influencing the anticipated therapeutic outcome. Typically, a substantial reduction in impurities is observed following the initial round of ultracentrifugation, with enhanced purity achieved through two to three subsequent cycles of density gradient ultracentrifugation (Khaparde et al., 2025; Lorek et al., 2025).

Ultracentrifugation faces serious scalability limitation due to the difficulty in handling larger volumes. Hence, scalability becomes relatively easy due to the flow-based nature of chromatographic techniques (Khaparde et al., 2025; Lorek et al., 2025).

Nevertheless, recent advancements in continuous flow ultracentrifugation through Alfa Wassermann AW Promatix 1,000 reported a recovery rate exceeding 55% in a single run, demonstrating the capacity for linear scalability with rotors accommodating up to 50 L, thereby meeting industrial-scale requirements (Khaparde et al., 2025; Lorek et al., 2025).

##### 1.3.2.4 Polishing chromatography step

The main aim of ion-exchange chromatography purification step is to separate the empty and full capsids based on the difference in their surface charge distribution (Danaher Life Sciences, 2025).

Recent advancements in AAV vector enrichment by ion-exchange chromatography have focused on improving the separation of full and empty capsids, scalability, and process robustness.

- A notable innovation is the “two-pass” anion-exchange strategy, which uses sequential micro step conductivity increases followed by re-chromatography of enriched fractions, acheived higher full capsid enrichment across multiple serotypes at production scale (Huato Hernandez et al., 2024).
- Membrane-based ion-exchange chromatography methods, such as Mustang Q and other anion-exchange membranes, have been optimized using DOE to define operating spaces that consistently yield over 70%–80% full capsids, with robust performance across different serotypes and feed streams, and demonstrated scalability to manufacturing levels (Lavoie et al., 2023; Huato et al., 2024).
- Dual salt elution gradients and stepwise conductivity increases have also been shown to enhance resolution and purity, with dual salt approaches have achieved up to 75% full capsids and significant aggregate reduction (Dickerson et al., 2021; Hejmowski et al., 2022).
- The use of isocratic elution methods reported an advantage in buffer consumption and process robustness, providing a simpler and more scalable alternative to traditional gradient elution (Meierrieks et al., 2025).
- Combining steric exclusion chromatography with ion-exchange chromatography has enabled serotype-independence, high-purity AAV vector purification in fewer steps, which improved the overall process efficiency and scalability (Meierrieks et al., 2025).

#### 1.3.3 Formulation, fill-finish and storage operations

Formulation, fill-finish, handling and storage become the most critical segment of the manufacturing process (Figure 2). The stakes are high and handling the final formulation where the integrity of the product is preserved during shell-life becomes challenging as these formulations possess complex molecular structures and various factors affect physicochemical properties of the final formulation (Srivastava et al., 2021).

##### 1.3.3.1 Formulation

Formulation challenges include the physical, chemical and immunological stability of the AAV vectors which include aggregation, denaturation and interaction with light, container closure system, loss of product integrity during shelving at ultra-low temperatures and maintaining the product storage at ultra-low temperature during product logistics from manufacturing sites to clinical trial facilities (Croyle et al., 2001; Srivastava et al., 2021).

Here are some recently reported advancements in formulation, which have shown enhanced stability of viral preparations during storage and shipping. The use of distinct buffer systems and cryoprotectants has been demonstrated to significantly improve the stability of viral preparations throughout the processes of storage and transportation (Srivastava et al., 2021; Elizabeth Mann, 2025).

##### 1.3.3.2 Fill-finish operations

The implementation of adaptable modular platforms facilitates the aseptic filling and closure of diverse container types utilizing a singular apparatus. Sophisticated robotic systems can be engineered for environments compliant with GMP Class A standards to manipulate various container types while eliminating any risk of contamination and the use of automated closed systems can effectively segregate the product from the external environment, thus attenuating contamination risks and decreasing the requirements for cleanroom conditions (Srivastava et al., 2021; Elizabeth Mann, 2025). An integrated automation strategy can combine multiple stages into a singular automated workflow, encompassing processes from formulation to packaging and enhanced in-line product monitoring ensures 100% in-line process oversight and real-time surveillance, thereby securing consistent product quality and diminishing the incidence of manufacturing failures (Srivastava et al., 2021; Som et al., 2024).

## 2 Process characterization

Overcoming the challenges in the manufacturing space requires a deliberate shift from empirical approaches to science-driven, data-informed strategies at every stage of the process. So, the rapidly growing advancements reported in the domain can propel the manufacturing success by employing tailor-made strategies to the chosen process and by making science-backed decisions at every step, whether it is cell line selection, transfection optimization, harvest timing, or purification strategy. Thus, manufacturers can build robust, scalable, and reproducible processes. This not only facilitates regulatory compliance but also accelerates the path from cell culture to care, ultimately benefiting patients with safer and more effective gene therapies.

The dynamic viral vector systems and the intrinsic biological variability present in the production processes are sensitive to handling and can affect the process parameters at various stages of manufacturing. This process inconsistency can lead to the design of processes with less confidence and with no controls. So, to build process with control, a design space needs to be established through rigorous experimentation and statistical analysis. This helps in identifying the critical process parameters (CPPs) and their relationship to critical quality attributes (CQAs). This approach can help the manufacturer to achieve quality at every stage of the process where process consistency can be maintained, even under variable conditions. Thus, process characterization plays an important role in designing and optimizing process to robustness. Moreover, process characterization supports risk-based approaches to process validation, facilitates root cause analysis in the event of deviations, and lays the foundation for continuous process improvement. Ultimately, it serves as a cornerstone for building a well-controlled, scalable, and compliant manufacturing platform for AAV-based gene therapies.

Process characterization is a critical step in the manufacturing of AAV-based gene therapies, serving as the foundation for ensuring product quality, safety, and consistency. Through systematic evaluation of process parameters and their impact on CQA, manufacturers can optimize production, minimize variability, and meet increasingly stringent regulatory requirements. This approach not only supports efficient scale-up but also establishes a robust framework for process validation, subtle process improvements and successful commercialization of innovative gene therapy products (Alliance for Regenerative Medicine, 2021).

**FIGURE 3:**
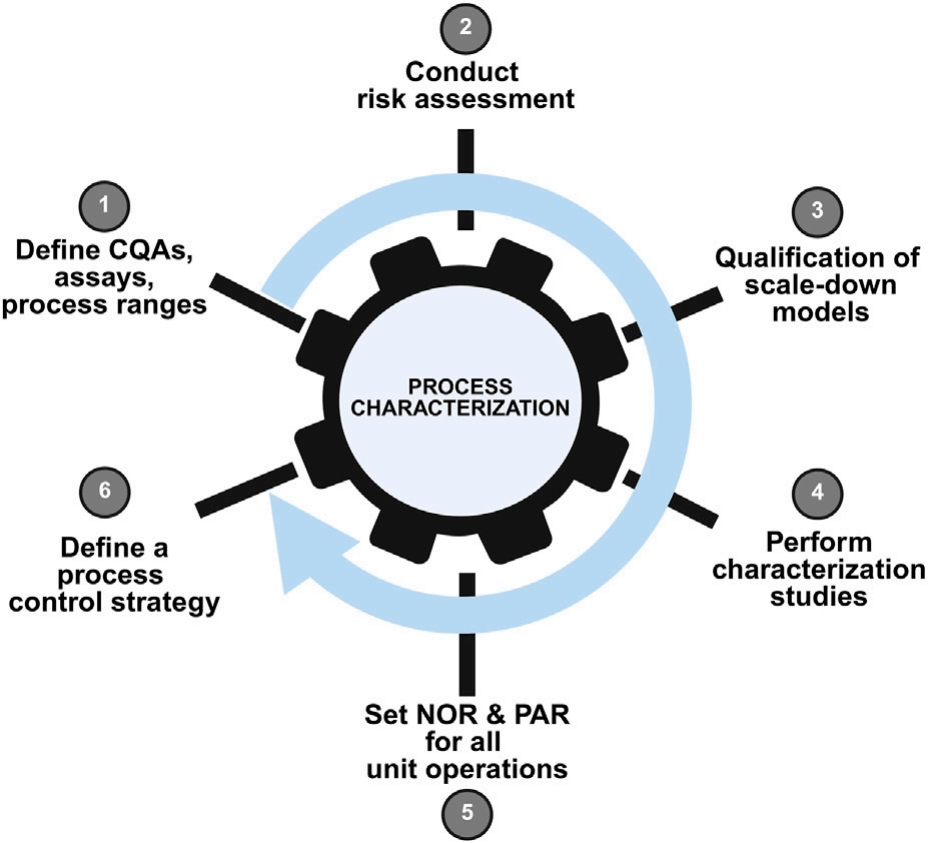
Schematic representation of a typical process characterization workflow. This figure depicts the systematic approach to process characterization in AAV manufacturing, encompassing identification of critical process parameters (CPPs) and critical quality attributes (CQAs), experimental design using statistical methods (e.g., Design of Experiments), data collection, and multivariate analysis. The workflow demonstrates how process understanding is built to support control strategy development and process validation.

Process characterization prepares for process validation, product license application, and commercialization. As an essential milestone in a product’s lifecycle, process characterization benefits from a systematic, standardized strategy aligned to a quality-by-design approach. Before drugs products are into the market, process characterization data has become the most concerned part of regulatory agencies because it serves as evidence to prove that the manufacturer can consistently deliver a quality product. An outline of an overall process characterization strategy workflow is provided in Figure 3.

### 2.1 Process characterization workflow

Process characterization is a set of experiments on a small scale, in which operational parameters are purposely varied to determine their effects on product quality attributes and process performance. The results from the process characterization studies are used to determine the process performance qualification (PPQ) ranges and acceptance criteria and a robust process control strategy (Alliance for Regenerative Medicine, 2021).

Process characterization is based on risk assessment and is a part of quality risk management. During the initial stages of process characterization, potential critical process parameters (pCPPs) are defined for each operation unit. The acceptable ranges of all high–risk parameters must be defined in the subsequent process characterization studies, and it must be confirmed that these ranges will not have any adverse impact on CQA (Alliance for Regenerative Medicine, 2021).

#### 2.1.1 Risk assessment

In the risk assessment (Figure 3), the CQA of the drug product is defined using failure mode effects analysis (FMEA) data or the cause and effect (C&E) matrix. Then the potential CPP in upstream, downstream, and formulation processes were identified based on the historical data (quality target product profile, chemistry, manufacturing and controls data, clinical data and paper reports) from manufacturing process and knowledge in and evaluated in design of experiment (DOE) approach (Alliance for Regenerative Medicine, 2021).

#### 2.1.2 Failure mode effectiveness analysis

A risk score can be calculated by considering each parameter’s severity, possibility, and detectability. Then, using the risk ranking table, identify the priority of risk control, which is then used to evaluate the product’s potential CQA as well as the CPP that may affect its quality (Alliance for Regenerative Medicine, 2021).

#### 2.1.3 Cause-and-effect matrix

A cause-and-effect matrix, also known as a prioritization matrix, is used to identify and prioritize key process input variables (process parameters) based on their impact on key outputs (quality attributes). It helps to understand which process inputs have the most significant influence on outputs, allowing for focused efforts on areas that drive quality improvement (Alliance for Regenerative Medicine, 2021).

#### 2.1.4 CQA evaluation

**FIGURE 4:**
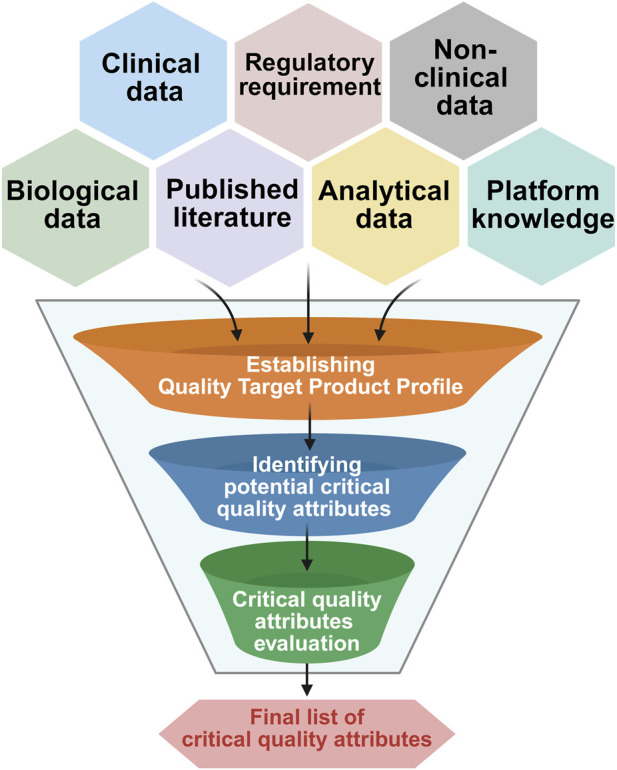
Generalized workflow for critical quality attribute (CQA) evaluation. This diagram outlines the stepwise process where different data sources were considered to define quality target product profile followed by potential CQA identification and finalizing CQA with acceptance criteria establishment.

The technical understanding of the drug product and historical data helps us to define the CQAs through risk assessment. This marks the beginning of the process characterization study. The CQAs are mainly derived from the quality target product profile (Alliance for Regenerative Medicine, 2021). The general process flow for CQA evaluation is described in Figure 4.

#### 2.1.5 CPP evaluation

**FIGURE 5:**
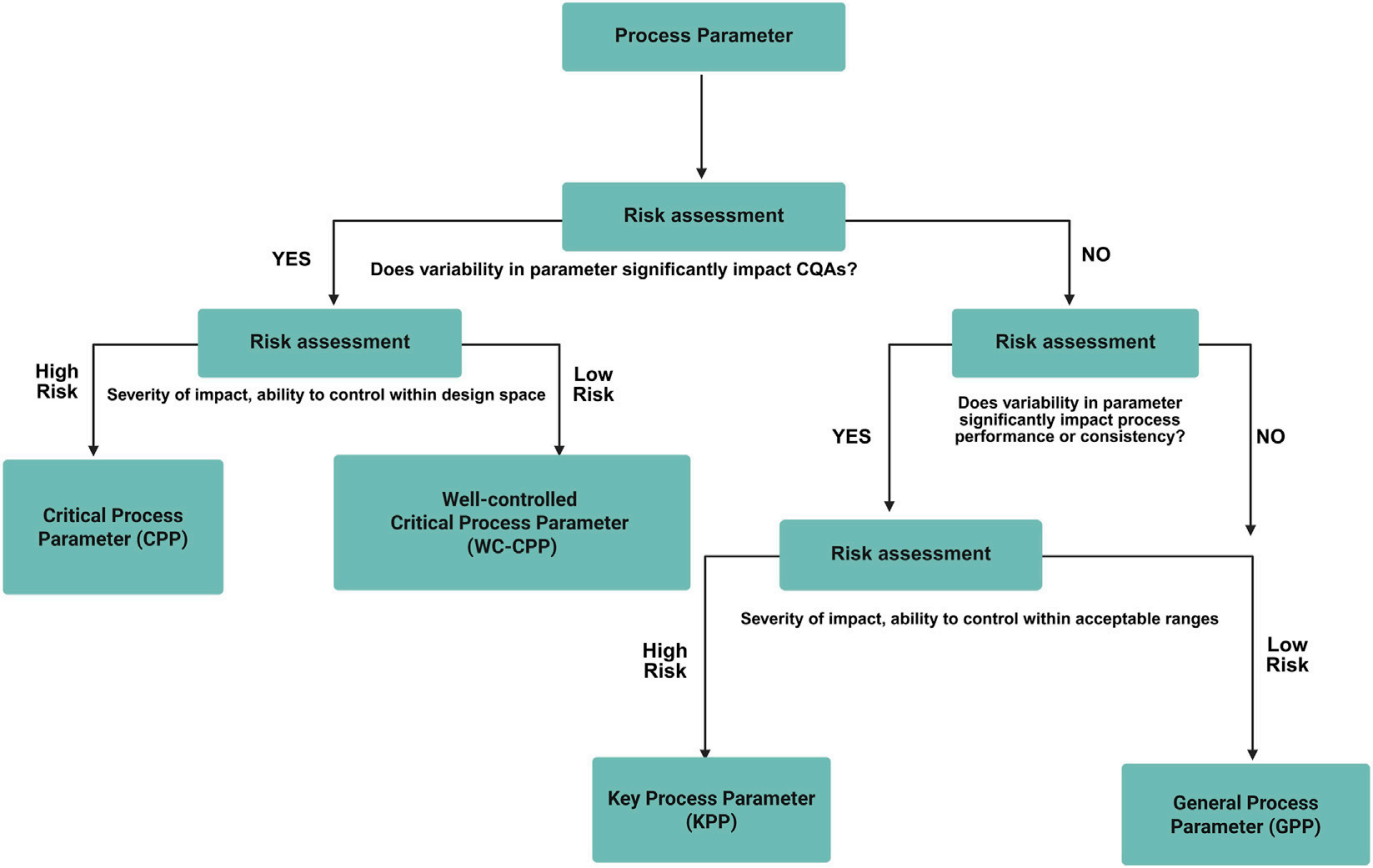
General workflow for critical process parameter (CPP) evaluation. The figure presents the structured approach for identifying, assessing, and ranking process parameters that influence product quality. It highlights the link between process parameters and CQAs through risk assessment to establish parameter criticality and design space boundaries.

Based on the CQA list, risk assessment tools (such as FMEA and C&E matrix) are used to eliminate process parameters which are considered non-critical for the identified process parameters and all the pCPPs were identified and studied for their effects on the process to develop a control strategy (Alliance for Regenerative Medicine, 2021). The general process flow for CQA evaluation is described in Figure 5.

#### 2.1.6 Scale-down model (SDM)

An SDM is built to represent large-scale manufacturing and utilizes them to study all CPPs so that multiple experiments can be performed. Key process performance parameters must be similar between the commercial scale and the SDM under the defined process. The larger the scale–down factor, the more efficient the process is.

The core of SDM development involves matching critical engineering parameters between scales to ensure equivalent cellular environments. To achieve similar mixing conditions, a constant power per unit volume (P/V) is maintained while oxygen transfer coefficient matching ensures adequate oxygen supply for cell growth and AAV vector production (Flickinger, 2009). Mixing time equivalence is established to maintain homogeneous conditions, and shear stress levels are controlled to prevent cell damage during the production process. Experimental qualification involves running parallel batches at both scales, comparing cell growth kinetics, metabolic profiles, AAV vector titers, and product quality attributes. Statistical validation using methods such as multivariate data analysis (MVDA), principal component analysis (PCA), and equivalence testing (such as Two One-Sided Test - TOST) confirms that the SDM adequately represents the commercial process. If equivalence is not achieved, parameters are iteratively adjusted until acceptable correlation is demonstrated, followed by documentation of the qualified model for regulatory submissions and routine use in PC studies (Flickinger, 2009; Parenteral Drug Association, 2013).

This systematic approach ensures that the SDM serves as a reliable predictive tool for process optimization, troubleshooting, and regulatory compliance while significantly reducing development timelines and costs associated with AAV-based gene therapy manufacturing (Flickinger, 2009; Parenteral Drug Association, 2013). Some critical unit operations such as bioreactor design and chromatography purification can be demonstrated using SDM to understand the role of process parameters (Alliance for Regenerative Medicine, 2021).

##### 2.1.6.1 SDM for a commercial-scale bioreactor system

**FIGURE 6:**
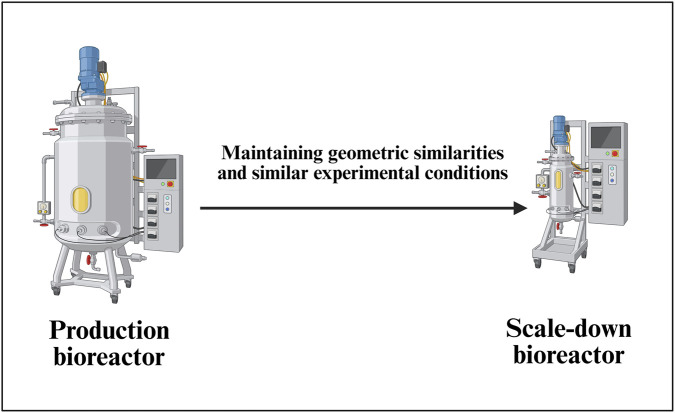
Development of a scale-down model (SDM) for a commercial-scale bioreactor. This schematic demonstrates the principles of scale-down model establishment for AAV upstream processes, ensuring representativeness of critical parameters such as mixing, mass transfer, shear stress, and transfection efficiency. The SDM serves as a predictive tool for process characterization, optimization, and troubleshooting prior to full-scale manufacturing implementation.

Establishing a robust scale-down bioreactor model for AAV vector production (Figure 6) involves a systematic approach as it is essential for efficient process development, characterization, and troubleshooting while minimizing labor, risk of contamination and costs associated with large-scale operations (Alliance for Regenerative Medicine, 2021). A general scale-down bioreactor design is depicted in Figure 6.

The SDM selection process starts with comprehensive characterization of the target commercial-scale bioreactor system, typically ranging from 50L to 2000L for AAV vector manufacturing. CQAs must be clearly defined, including AAV vector titer (genome copies/mL), capsid integrity, full-to-empty particle ratio, and cell viability profiles. Key process parameters such as mixing conditions, oxygen transfer rates, pH control, and temperature profiles are mapped to understand their impact on product quality and yield. The scale-down platform selection considers geometric engineering principles, with typical volume ratios ranging from 1:10 to 1:100, using 2–3 L stirred-tank reactors (Flickinger, 2009; Parenteral Drug Association, 2013).

##### 2.1.6.2 SDM for a commercial-scale column chromatography set up

**FIGURE 7:**
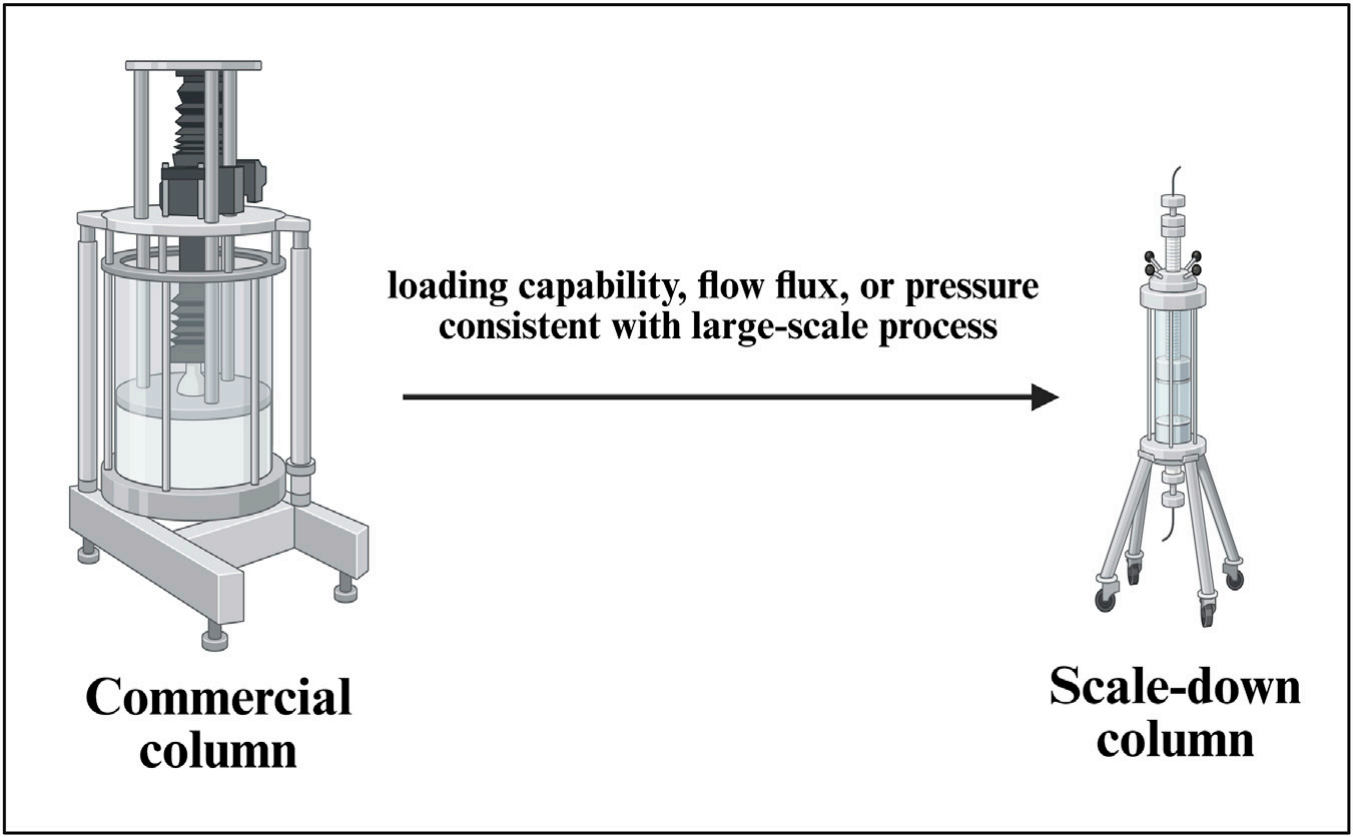
Development of a scale-down model (SDM) for a commercial-scale chromatography column setup. This figure illustrates the development and qualification of SDMs for downstream purification, capturing key variables such as resin bed height, linear velocity, residence time, and load density. The model facilitates robust evaluation of chromatographic performance, enabling predictive control of product purity, yield, and impurity clearance across manufacturing scales.

The SDM for a commercial-scale column chromatography set up (Figure 7) is considered with a with a linear scaling down of the parameters such as retention time and column height, same as the large-scale manufacturing process. In the mixing step, measures such as consistent power per unit volume (P/V) are applied to ensure that the mixing effect is the same as the commercial process. In the filtration step, a linear scaling down of process parameters is considered, keeping the loading capability, flow flux, or pressure consistent with the large-scale manufacturing process (Parenteral Drug Association, 2013). A general SDM for a column chromatography set up is depicted in Figure 7.

#### 2.1.7 Comparability study

Once the SDM is established, they are verified by comparing multiple runs at small scale to full-scale batches using a combination of proportional and identical process parameter values (refer Table 2) (Parenteral Drug Association, 2013). Generally, the comparability criteria are set as three standard deviations (or 95% confidence interval) according to manufacturing data. If the obtained product quality data lies within the set acceptable range, then the established SDM is considered reliable and can be regarded to represent the commercial processes (Parenteral Drug Association, 2013; Alliance for Regenerative Medicine, 2021).

**TABLE 2:** Scale-down variables.

Unit operation	Constant variables	Proportional variables
Bioreactors	Power per unit volume (P/V); liquid-phase mass transfer coefficient (k_L_a); temperature (T)	Revolutions per minute (rpm); Impeller diameter (D_i_); Volume (V); tank diameter (D_t_); superficial gas velocity (V_s_)
Chromatography columns	Bed height (L); protein load (g/L resin); linear fluid velocity (VF); residence time (t_R_); wash volumes	Column inner diameter (ID); volumetric flow rate (F); column volume (CV)

#### 2.1.8 Experimental design

DOE is a systematic method to study the relationship between multiple input and key output variables. During the process characterization study, the CPPs should be characterized by DOE approach and the control range of CPP should be defined to validate the accepted ranges of parameters in the commercial scale and to develop a robust control strategy. Common types of experimental designs are listed below (Alliance for Regenerative Medicine, 2021).

1. Fractional factorial design: This design can screen out the main effects with fewer experimental runs but cannot identify interactions and quadratic effects.
2. Full factorial design: This design allows for estimation of main effects and interactions. But this method requires many tests, and quadratic effects may be ignored.
3. Response Surface Methodology: This method adds to factorial design where the main effects, interaction effects, and quadratic effects were identified. But the number of experiments that are run is so large, which is only suitable for analyzing three and four factors.
4. Definitive Screening Design: This design uses three levels of continuous factors and estimates of main effects, some interactions and quadratic terms. From the results of the screening study, the parameters with main effects are confirmed and if the interaction effects or quadratic effects are found significant, then response surface studies are required to quantify those effects and then the design space is defined based on the experimental data.

#### 2.1.9 Control strategy

**FIGURE 8:**
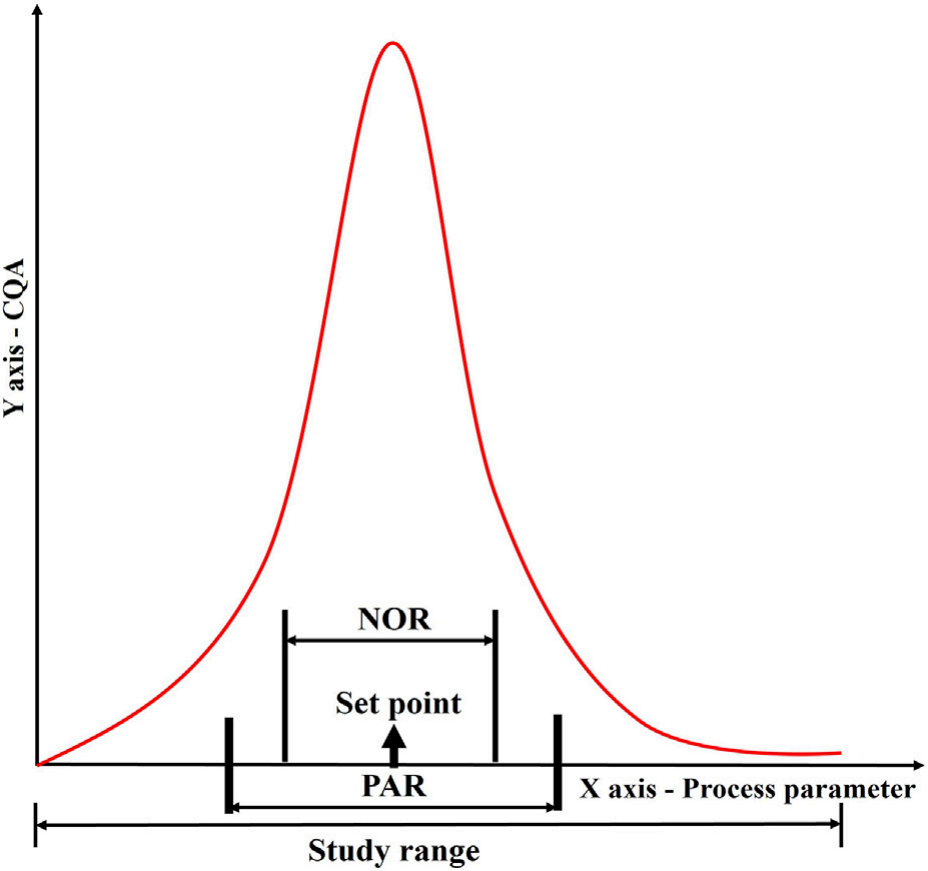
Establishment of a control strategy through determination of normal operating range (NOR) and proven acceptable range (PAR) for a given process parameter in relation to a CQA. This figure illustrates the correlation between process parameters and product quality, showing how data-driven approaches define operational boundaries. The graph depicts how NOR and PAR are derived from experimental and historical data to ensure robust process performance and product consistency within validated limits.

Based on the results of the DOE, a design space is defined. The proven acceptable range (PAR) of parameters is defined from the design space. Keeping other parameters constant, the given parameter varies within PAR to produce quality products (Figure 8) (Alliance for Regenerative Medicine, 2021).

The NOR is a range of process parameter values that contains common operational variability that cannot always be controlled. It is established for various process parameters of the same process step and is not intended to introduce flexibility in the conditions for manufacturing. The NOR is presented in marketing authorizations as what is achievable. The NOR is defined based on the optimal operating conditions that result in the desired product quality, yield, and process efficiency. It can be refined within the confines of PAR by comprehensive consideration of the set point, equipment, and process variability. The PAR, on the other hand, is the range of process parameter values that have been scientifically demonstrated to consistently produce a product that meets the specified quality attributes (Alliance for Regenerative Medicine, 2021).

The PAR is determined through robustness studies in which the process is deliberately subjected to variations in the input parameters to determine the effect on product quality. The PAR is often wider than the NOR, as it accounts for potential process variability and allows for flexibility in manufacturing operations. During commercial manufacturing, once a parameter exceeds PAR/NOR, it indicates the batch failure. Variations within the design space will not be regarded as change, and manufacturers need not submit supplementary applications to their regulatory agency (Alliance for Regenerative Medicine, 2021). The relationship between PAR and NOR is depicted in Figure 8.

### 2.2 Process characterization in AAV vector manufacturing: CQAs and analytical challenges

In AAV vector manufacturing, the process characterization centers on defining and controlling CQAs that impact product safety and efficacy. These CQAs encompass fundamental structural and functional properties that must be continuously monitored throughout the manufacturing process to ensure consistent product quality. The ideas from QbD principles along with risk-based approaches will guide manufacturers in identifying the process or product-related CQAs (Figure 4). Studies have identified quality attributes such as virus titer, capsid content and aggregation as potential CQAs which affect the purity, potency and safety of the drug product and efforts are being made to explore other potential CQAs (Tanaka et al., 2020; Gimpel et al., 2021; Kontogiannis et al., 2024).

The current methods of analytical process development in practice face hurdles in terms of lengthy turnaround time for analysis with low throughput (Gimpel et al., 2021). There is a lot of scope for researchers to develop and streamline rapid and high-throughput analytical methods which can quantify the CQAs. Since every AAV vector product under R&D phase is of distinct gene construct, the product or the process-related quality attributes will be highly specific to the manufacturing conditions and the key starting materials used in process. Hence it becomes a niche area for researchers and manufacturers to have robust manufacturing process development and the corresponding analytical data from the state-of-the-art techniques which can help manufacturers gain better process confidence and also in the establishment of a solid control strategy.

## 3 Process validation

**FIGURE 9:**
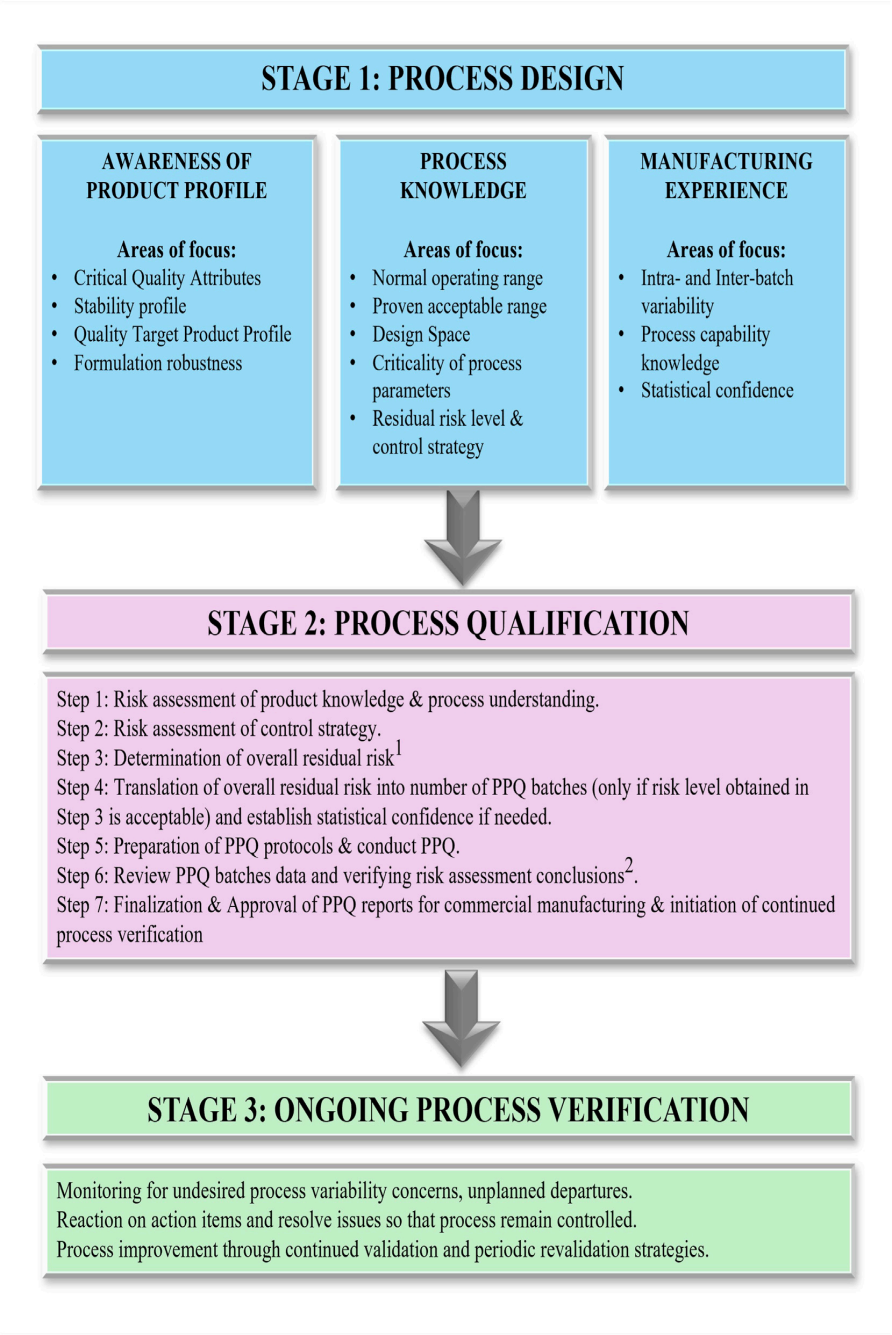
Comprehensive workflow of activities encompassing the three stages of process validation: process design, process qualification, and continued process verification. This figure provides a structured view of process validation for AAV manufacturing, detailing the progression from initial process design and risk assessment (Stage 1) through process performance qualification (Stage 2) and ongoing process verification (Stage 3). Iterative feedback loops are included: (1) If the risk level is deemed unacceptable, the workflow returns to Stage 1 for further mitigation. (2) If PPQ criteria are not met based on risk level outcomes, the process reverts to earlier steps (1–5) for refinement. This closed-loop framework supports a lifecycle approach to validation and continuous improvement.

Process validation provides sound scientific evidence for performing the drug product manufacturing processes within defined parameters. It provides a logical basis that the overall process can be operated efficiently and consistently to produce a drug product that fulfills its predetermined specifications. The US FDA guideline outlines the expectations for process validation in a sequence of 3 stages (Figure 9), i.e., process design, process qualification, and ongoing process verification, respectively (U.S. Department of Health and Human Services Food and Drug Administration, 2011; Rathore and Sofer, 2012).

1. Process design (Stage 1) activities include the design of the commercial manufacturing process that will be reflected in planned master production and control records. The purpose is to achieve process consistency to deliver a product that meets its quality attributes.
2. Process qualification (Stage 2) involves the evaluation of process design for its robustness involving elements of facility design, equipment and utilities qualification and process performance qualification (PPQ).
3. Ongoing process verification (Stage 3) assures that the process is controlled (the validated state) during manufacture. The objective is to maintain a system to detect unplanned departures, undesired process variability, problems and determine whether action must be taken to correct, anticipate, and prevent problems so that the process remains in control. The generalized workflow for process validation is shown in Figure 9.

### 3.1 Risk-based justification for the number of PPQ batches

Due to the limited number of batches produced during development and early commercial phases, a risk-based and statistically justified process validation approach is essential for AAV-based gene therapy manufacturing process. The overall residual risk associated with any manufacturing process depends on three factors, i.e., product knowledge, process understanding, and large-scale/clinical manufacturing experience. The overall residual risk will be evaluated based on process impact score, risk priority number, or qualitative assessment based on overall product, process, and manufacturing knowledge, process variability, and control strategy is developed. Then the overall residual risk will be translated into the required number of PPQ batches. There are no industry standards or harmonized opinions to determine and justify the number of PPQ batches (Colandene et al., 2020). The minimum number of PPQ required can be determined based on the variability observed across different parameters in the previous campaigns of the manufacturing process. For example, if the manufacturing process is tightly controlled, fewer batches can give statistically meaningful data. However, if the variability observed in the process is large, then multiple campaigns need to be performed to show the robustness of the process. As of the current scenario, there were no harmonized standards or specific guidance that recommends for requirement on the number of batches. However, three consecutive batches of PPQ are the recommendation for the process under control as process validation strategy (Colandene et al., 2020).

## 4 Discussion

As the manufacturing of AAV vectors accelerates, numerous important trends are emerging demonstrating the future of gene therapy. Together, these trends illustrate an encouraging outlook for AAV vector production, where personalized medicine, adherence to regulations, broader applications, collaboration, and streamlined processes come together to advance gene therapy. The worldwide AAV vector market is expected to expand at a compound annual growth rate of approximately 14% until 2035 (Rootsanalysis, 2023). Fueled by the increasing need for gene therapies and tailored medications, the AAV vector market is expected to expand more rapidly in the upcoming years.

There are ample opportunities and scope for improvement in the AAV vector manufacturing space where research has been progressing at a rapid pace in the development of continuous manufacturing over conventional processes. Studies have been reporting an enhancement in viral vector yield translating better process efficiency (Benskey et al., 2016; Gutiérrez-Granados et al., 2018; Yang et al., 2023; Park D. et al., 2024). Such innovations can lead to the development of multiple gene therapies in an accelerated timeline and can transform AAV-based gene therapies into a plug and play mode by making it easily accessible to patients with ultra-rare conditions across the world.

Through a rigorous risk-based approach, process characterization for AAV-based gene therapies unites CQAs with data-driven mapping of potential CPPs. Leveraging well-validated SDM and strategic DOE, manufacturers establish PAR and NOR that fortify an end-to-end control strategy. Once the process confidence is achieved, it is validated by a set of guidelines so that desired product quality is achieved at the end of the manufacturing process ensuring that the quality is maintained throughout the manufacturing process through process validation. This disciplined methodology safeguards consistency in potency, purity, and safety from laboratory to commercial scale, smooths the path to process validation and licensure, and ultimately enables reliable and efficient delivery of life-changing gene therapy products.

The future of AAV capsid design lies in the development and utilization of advanced analytical technologies such as cryoelectronic microscopy, mass spectrometry–based proteomics, molecular dynamics simulations, and custom-built AI tools. These state-of-the-art techniques coupled with AI and predictive modeling can have a significant impact in explaining the capsid behavior, post-translational modifications, host interactions, capsid functionality across varied environments, production methods, buffer conditions, and host species (Suarez-Amaran et al., 2025). This will empower researchers to engineer AAV capsids optimized for enhanced stability, immune evasion, targeted tissue delivery, and therapeutic effectiveness. Artificial Intelligence is poised to play a central role in the next-generation of advanced manufacturing technologies, transforming key aspects of industry. In design optimization, AI can predict optimal recombinant protein and gene therapy sequences to enhance expression, safety, and yield. It enables predictive process modeling to simulate manufacturing outcomes and inform proactive adjustments. AI also supports process optimization and automation through integration with closed, modular systems, improving sterility, reducing variability, and shortening time-to-release. Additionally, AI enhances advanced analytics and process control by enabling real-time monitoring and adaptive control systems (Suarez-Amaran et al., 2025).

Various challenges were faced by the AAV vector manufacturers due to time-consuming and expensive analytical methods which provide limited real-time insights during process monitoring. The industry needs experts to conduct value-driven research to bridge the gap in attaining manufacturing process control for AAV vectors. A study has outlined the implementation of AI-driven soft sensing with predictive modeling which can help in real-time estimation of CQAs including viral titer, capsid integrity, and process performance indicators from readily available process variables. This capability can transform manufacturing control by providing continuous process monitoring, early deviation detection, and predictive quality assessment throughout the production campaigns (Iglesias et al., 2023).

Recent work in mechanistic modeling of empty-full capsid separation using anion-exchange membrane chromatography showed that AI-enhanced models could predict recovery yields with remarkable fidelity and identify process conditions leading to significant enrichment of functional capsids. This represents an early case study of *de novo* mechanistic modeling application for full capsid enrichment in AAV vector manufacturing, demonstrating AI’s potential in downstream process development (Gomis-Fons et al., 2024).

As many key industry players have been attempting to harness the therapeutic potential of the AAV-based gene therapies, the utmost priority is to standardize the orthogonal analytics and the accessibility of traceable reference AAV vector compounds for researchers to achieve robust analytical method development in their manufacturing space. There is a definite need for benchmarking in the variability of potency and genome titer assays, and it could be harmonized by conducting multi-laboratory ring trials.

The researchers should actively focus on how upstream expression stoichiometry could quantitatively influence the intermediate capsid formation so that the resultant upstream design can reduce the work-up during purification step. A real-time comparison needs to be drawn while choosing multi-column chromatography vs. ultracentrifugation/gradient approaches on factors like cost of raw materials, scalability, and impurity clearance. The manufacturers should invest their time, resources and qualified personnel to utilize process analytical technology which would enable lot-by-lot comparability.

Underlining the importance of patient safety and accelerating the approvals, the regulatory agencies should be equipped with a standard set of orthogonal assays (identity, genome titer, infectivity/potency, empty/intermediate/full quantification) and transparent acceptance criteria tied to clinical-dose rationale. This can bring AAV vector manufacturing standards closer to accessible and safer biologics to patients while recognizing vector-specific complexities.

In anticipation of future advancements, automated platforms that integrate high-throughput screening with synthetic biology will permit the development of fully customizable AAV vectors designed for specific diseases or tailored to individual patients through personalized medicine. In addition, the innovative approaches that can address multiple diseases by a single gene could be a quantum leap for AAV-based gene therapies. Such ideas can revolutionize AAV vectors into a remarkably precise and adaptable instrument, establishing new benchmarks for the forthcoming era of gene therapy. Further, the successful integration of emerging technologies in manufacturing with clear regulatory frameworks and harmonized guidelines for AI-driven decision-would help achieve the goal of advancement for small and mid-sized manufacturers.
